# Does metformin usage improve survival in head and neck squamous cell carcinoma? A population-based study

**DOI:** 10.1186/s40463-018-0322-7

**Published:** 2018-12-04

**Authors:** Alexandra E. Quimby, Nicole L. Lebo, Rebecca Griffiths, Stephen Hall, Jim Dimitroulakos, Stephanie Johnson-Obaseki

**Affiliations:** 1 Department of Otolaryngology, Head and Neck Surgery, University of Ottawa, 501 Smyth Rd, Module S - Room M2566, Box 216, Ottawa, ON K1H 8L6 Canada; 2 Institute for Clinical and Evaluative Sciences, Queen’s University, Abramsky Hall, Room 208, 21 Arch Street, Kingston, ON K7L 3N6 Canada; 3 Department of Otolaryngology, Head and Neck Surgery, Queen’s University, 18 Barrie Street, Kingston, ON K7L 3N6 Canada; 4 Ottawa Hospital Research Institute, 501 Smyth Rd, Box 511, Ottawa, ON K1H 8L6 Canada

**Keywords:** Squamous cell carcinoma, Metformin, Larynx, Hypopharynx, Nasopharynx

## Abstract

**Background:**

We sought to expand upon preliminary data suggesting that metformin confers a survival benefit to patients with head and neck squamous cell carcinoma (HNSCC).

**Methods:**

A large-scale retrospective cohort study of all patients in Ontario diagnosed with squamous cancer of the larynx, hypopharynx, and nasopharynx between Dec 1st 2007 to Dec 1st 2012 was undertaken. The Institute for Clinical and Evaluative Sciences was accessed to obtain patient demographic, treatment and outcome information. We included patients on metformin at the time of diagnosis. Kaplan Meier methods and Cox Regression models were used.

**Results:**

Patients taking metformin at the time of diagnosis had a higher comorbid status but were otherwise similar to patients without metformin usage. Using multivariate analysis, neither overall survival nor disease specific survival was improved in patients on metformin (OS: HR 1.123, *p* = .338; DSS: HR 1.048, *p* = .792).

**Conclusions:**

No survival advantage was observed in patients with HNSCC taking metformin at the time of diagnosis.

**Electronic supplementary material:**

The online version of this article (10.1186/s40463-018-0322-7) contains supplementary material, which is available to authorized users.

## Introduction

Head and neck squamous cell carcinoma (HNSCC) accounts for approximately 6% of all new cases of cancer in Canada [1, 2]. Over the past two decades, Human Papillomavirus (HPV) has emerged as an independent etiologic factor for HNSCC. As a result, epidemiologic trends have shifted towards increased incidence, younger age at diagnosis, and overall improved survival of HNSCC [3]. However, outcomes for patients with non-HPV associated HNSCC remain poor. Treatment of HPV-negative HNSCC can be challenging, and is met with considerable patient morbidity. For cancers of the larynx, hypopharynx, and nasopharynx – three subsites in which HNSCC is uncommonly attributed to HPV infection – treatment can consist of either primary surgery (in the case of larynx and hypopharynx), primary radiotherapy (early laryngeal cancers), or chemoradiotherapy (nasopharynx or advanced larynx/hypopharynx). Surgical morbidity at these sites can be high, and toxicities from chemoradiotherapy are common, and can significantly impact patients’ quality of life [4–7].

There has recently been an impetus for the discovery of effective targeted, novel adjuvant therapeutic agents for the treatment of HNSCC [8]. Epidermal growth factor (EGFR) inhibitors (cetuximab, gefitinib, erlotinib) have demonstrated early promise in the treatment of HNSCC [9–13]. The use of statins has also recently been shown to improve overall- and disease-specific survival in patients with HPV-negative HNSCC [14–16]. Combination novel therapeutics have also shown promise, for example, Monenisin – an animal antibiotic – has been shown to enhance the cytotoxicity of both erlotinib and lovastatin in vivo [17]. Despite the promise suggested by these targeted agents, though, they have made less of an impact on the survival outcomes in HNSCC to date than initially hypothesized. An ongoing need for the discovery of effective, targeted agents for the treatment of HNSCC exists.

Metformin is a biguanide oral antihyperglycemic used in the treatment of type 2 diabetes mellitus. Diabetics are known to have an increased risk of total body cancer, with hyperinsulinemia and hyperglyceimia proposed as carcinogenic mechanisms [18–20]. Metformin has insulin-sensitizing and glucose-lowering effects via a pathway that involves the activation of AMP-activated protein kinase (AMPK) [21, 22]. Additional downstream effects of AMPK target phosphorylation include inhibition of cellular growth and proliferation under stress conditions [23–25]. Through activation of AMPK in human carcinoma cells, metformin inhibits clonal survival in vitro, and enhances the effects of ionizing radiation on these cells [26]. It has been shown to enhance radiation effects on head-and-neck cancer cell lines in both in vivo and in vitro models [27]. Several observational studies in humans have also supported an anti-tumorigenic effect of metformin, correlating metformin use in diabetics with decreased cancer incidence and mortality, and improved treatment response in colorectal and breast cancers [28–34]. In patients with cancer of the larynx, a recent single-centre study demonstrated improved survival in diabetics taking metformin compared with diabetics not on metformin [35]. This preliminary evidence, taken together with the minimal side-effect profile of metformin, make it an appealing potential adjuvant treatment for HNSCC [36].

On the basis of this growing body of evidence, we conducted a large-scale retrospective cohort study to determine whether HNSCC patients taking metformin have a survival advantage compared with patients not on metformin. To account for the fact that HPV status is not routinely recorded for all HNSCC, and to control for the confounding effects of HPV-positivity on HNSCC survival, we selected only cases of HNSCC in subsites where HPV is rarely the predominant etiologic agent: the larynx, nasopharynx, and hypopharynx [4–6, 37, 38].

## Methods

### Data sources and patient population

A population-based retrospective cohort study was performed using data sources retrieved from electronic databases maintained at the Institute for Clinical Evaluative Sciences (ICES), where patient data is linked between databases using unique encoded identifiers.

The source of our patient population was The Ontario Cancer Registry, which includes all incident cases of cancer in Ontario. We identified all patients over the age of 65.5 diagnosed with nasopharynx, hypopharynx, or larynx malignancy between January 1, 2007 – December 31, 2012. We excluded patients previously diagnosed with HNSCC within 10 years prior to their present diagnosis date.

Our cohort was linked to the Ontario Drug Benefit Database (ODB) and divided into patients who were taking metformin at the time of diagnosis of HNSCC and those who had no metformin exposure. The ODB database includes claims from all patients eligible for the Ontario Drug Benefit Program. Eligibility is limited to those with a valid card for Ontario’s universal, publically-funded healthcare system – the Ontario Health Insurance plan (OHIP) – who are > 65 years of age, and patients enrolled in other specialized programs. We excluded control patients who had exposure to metformin within a year prior to or after their diagnosis. Primary analysis was conducted with metformin use being defined as the patient taking metformin at the time of diagnosis of malignancy. Secondary analyses were performed to assess whether duration of metformin use impacted results, with the definition of metformin use adjusted to, (a) metformin use for a minimum of 1 year prior to diagnosis and 4 months after diagnosis, and, (b) metformin use for a minimum of 1 month prior to diagnosis and 4 months after diagnosis. For secondary analyses, case and control patients that died within 4 months of their diagnosis were excluded.

### Covariates

Identified patients were classified into one of six treatment groups: 1) primary surgery with or without adjuvant radiation and/or chemotherapy, 2) primary radiation therapy (RT) with or without adjuvant/ salvage surgery, 3) primary combined chemoradiotherapy (CRT) with or without adjuvant/ salvage surgery, 4) induction chemotherapy before radiotherapy, 5) palliative/ no treatment, or 6) other therapies not captured by the aforementioned groups. Surgeries were identified through two means: The Canadian Institute for Health Information (CIHI) database, which contains all hospital discharge records, and The OHIP Claims database, which captures physician billing records. Patients were classified as having undergone primary surgery if they underwent pharyngo-laryngectomy or “total excision” of the glottis, supraglottis, or larynx, defined by the following ICD10 Procedure codes – 1GA89, 1GB89, 1GE89, 1GE91 – and OHIP fee codes- M081, S068 – within 4 months of diagnosis [39]. Glottic cancer patients who had partial surgeries or biopsies as their only treatment were also classified in the primary surgery group. Radiotherapy and chemotherapy treatments were abstracted from the Cancer Care Ontario’s Activity Level Reporting (ALR) Database. A patient was considered to have had curative radiotherapy if they were administered a total dose of ≥ 5000 centigray (cGy) in ≥ 20 total fractions to the relevant head and neck region within 4 months of diagnosis. A patient was categorized as receiving CRT if the chemotherapy started no more than 30 days prior to the initiation of radiotherapy. Any patient who received chemotherapy starting prior to this cutoff was classified in the induction chemotherapy group. Any patient who had RT to the head and neck at a palliative dose, received chemotherapy alone, or received no treatment at all within the first 4 months was classified in the palliative/ no treatment group. All other treatment regiments were classified as ‘other’. All patients classified as having undergone palliative/ no treatment, ‘other’ treatments, or induction chemotherapy before radiotherapy were excluded from survival analyses.

Comorbidity was calculated based on the Elixhauser Comorbidity Index [40–42]. Hospital admissions within 5 years prior to diagnosis, abstracted from the CIHI Database, were used to calculate the comorbidity score. The Elixhauser score ranges from 0 to 31 based on 31 possible comorbid diagnoses. All diagnosis types were included in the calculations. Patients were categorized as receiving a score of 0, 1, 2, or 3 + .

Demographic information for our cohort was retrieved from the Registered Persons Database (RPDP) supplied by the Ministry of Health and Long-term care. This dataset contains demographic information on anyone who has ever had an OHIP card number.

### Outcomes

The Office of the Registrar General Database (ORGD) was used to retrieve vital status and cause of death information to determine overall survival (OS) and disease-specific survival (DSS) for each patient. Information on vital status was available until March 31, 2015, and cause of death was available up to Dec 31, 2013.

### Statistical analysis

SAS Software (Version 9.4, SAS Institute, Cary, NC, USA) was the statistical software used. We compared the distribution of demographics, treatments, and site between metformin users and non-users using the chi-square (χ [2]) test for categorical variables and t-test/ANOVA (one-way analysis of variance) for continuous variables. Survival curves and log-rank scores were produced using the method of Kaplan and Meir [43]. To evaluate the survival benefit of metformin, we used Cox proportional hazards models to calculate hazards ratios (HR) with 95% confidence intervals (CI). Age, gender, treatment regimen, comorbidity, and cancer site were controlled for in multivariate models. All variables were analyzed as categorical variables. A *p*-value of < 0.05 was deemed statistically significant.

## Results

A total of 1679 patients (metformin users and non-users) were identified from the linked OCR and ODB databases. This included 208 patients taking metformin at the time of diagnosis of HNSCC in the sites under investigation, and 1471 matched control patients who had not had any exposure to metformin within at least 1 year before and after their date of diagnosis. A total of 448 patients (43 metformin users, 405 non-users) were excluded for having undergone no treatment, ‘other’ treatments, or induction chemotherapy followed by radiation. After these exclusions, there were a total of 1231 patients. Of these, there were 165 taking metformin at the time of diagnosis of HNSCC, and 1066 who had not had any exposure to metformin within at least 1 year before or after their date of diagnosis. Demographic and clinical characteristics of all included patients (metformin users and non-users) are detailed in Table 1. As expected, Elixhauser comorbidity score was significantly different between those taking metformin at the time of diagnosis (namely, diabetic patients) and metformin non-users, with those taking metformin having significantly higher Elixhauser scores, indicating a greater average number of comorbid diseases. Otherwise, no significant differences existed between those using and not using metformin with regards to age, gender distribution, treatment type, and site of primary malignancy. Glottic larynx was the most common HNSCC subsite in both subgroups of patients.

**Table 1:** Demographic and clinical characteristics of metformin use and non-use groups

Variable	Category	Cases (Metformin at time of diagnosis)	“Controls” (No metformin for ≥ 1 year prior and ≥ 1 year after diagnosis)	*P*-value
N		165	1066	
Age				
	65–69	38 (23.0%)	280 (26.3%)	0.874
	70–74	49 (29.7%)	289 (27.1%)	
	75–79	44 (26.7%)	257 (24.1%)	
	80–84	25 (15.2%)	168 (15.8%)	
	85–90	6 (3.6%)	52 (4.9%)	
	= > 90	3 (1.8%)	20 (1.9%)	
Mean age at diagnosis (± SD)		74.55 ± 6.09	74.51 ± 6.35	0.934
Elixhauser Comorbidity Index Score				
	0	43 (26.1%)	637 (59.8%)	< 0.001
	1	40 (24.2%)	197 (18.5%)	
	2	30 (18.2%)	96 (9.0%)	
	3+	52 (31.5%)	136 (12.8%)	
Gender				
	Female	24 (14.5%)	175 (16.4%)	0.543
	Male	141 (85.5%)	891 (83.6%)	
Treatment type				
	Surgery+/−RT/CRT	31 (18.8%)	266 (25.0%)	0.058
	RT+/-Surgery	116 (70.3%)	646 (60.6%)	
	CRT+/-Surgery	18 (10.9%)	154 (14.4%)	
Primary site				
	Glottic larynx	105 (63.6%)	656 (61.5%)	0.406
	Hypopharynx	17 (10.3%)	152 (14.3%)	
	Nasopharynx	12 (7.3%)	55 (5.2%)	
	Supraglottic larynx	31 (18.8%)	203 (19.0%)	

*SD* Standard deviation, *RT* radiotherapy, *CRT* concurrent chemoradiotherapy

**Fig. 1:**
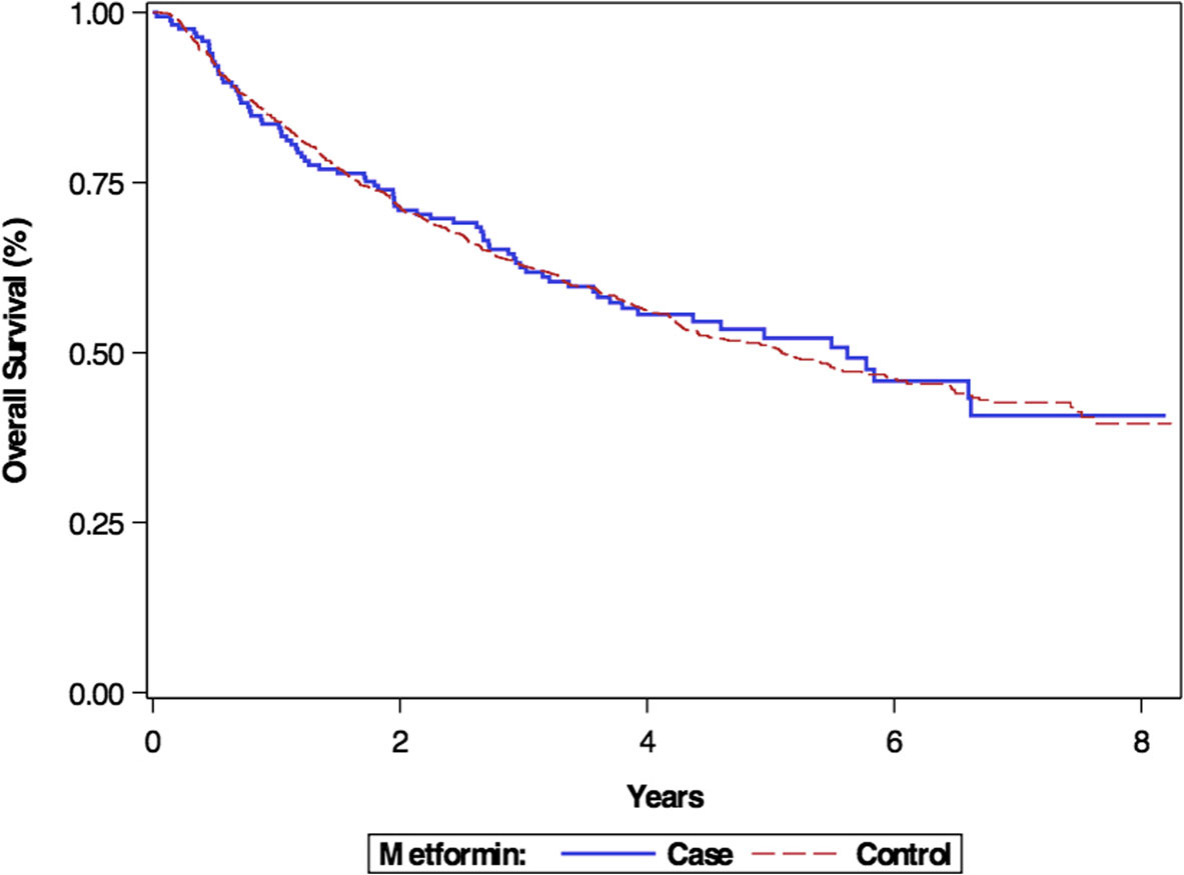
Kaplan Meier curve demonstrating overall survival of metformin users vs. non-users (*p* = 0.9182)

The Kaplan-Meier survival curve comparing OS between metformin users and non-users is displayed in Fig. 1. There was no significant difference in OS between patients taking metformin at the time of diagnosis and patients who had not had exposure to metformin for ≥ 1 year before and after their diagnosis of HNSCC (*p* = 0.9182).

**Fig. 2:**
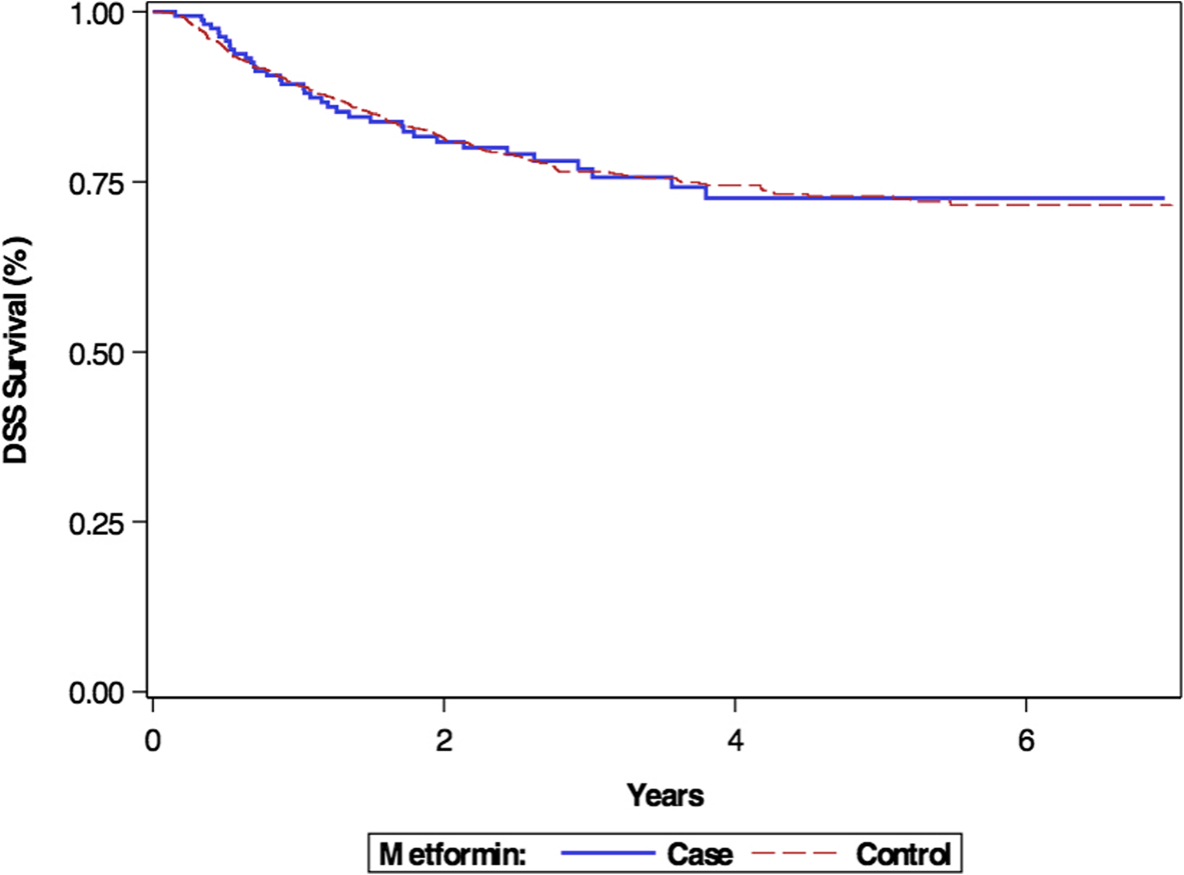
Kaplan Meier curve demonstrating disease-specific survival of metformin users vs. non-users (*p* = 0.9918)

The Kaplain-Meier survival curve comparing DSS for patients taking and not taking metformin at the time of diagnosis is displayed in Figs. 2. Similarly, there was no significant difference in DSS between metformin users and non-users (*p* = 0.9918).

Results from multivariate analysis of covariates for OS are summarized in Table 2. Covariates associated with significantly reduced OS included: increasing age – all age groups > 75 demonstrated a statistically significant decrease in OS compared with age group 65–69, with increasing hazard ratio (HR) for each increase in age group – Elixhauser comorbidity score of 3+ (HR 1.902, 95%CI [1.524–2.375]), and primary cancer of the hypopharynx or supraglottic larynx (hypopharynx: HR 2.933, 95% CI [2.336–3.683]; supraglottic larynx: HR 2.296, 95% [CI 1.867–2.825]) compared to patients with primary malignancy of the glottic larynx. Metformin use was not associated with a significant difference in OS compared to those not taking metformin (*p* = 0.4314, HR 1.104, 95% CI [0.863–1.413]).

**Table 2:** Multivariate regression analysis for overall survival (OS) in patients taking metformin at the time of diagnosis

Covariate	Category	Comparator	*P*-value	Hazard Ratio	95% CI
Age					
	70–74	65–69	0.2574	1.152	0.902–1.471
	75–79		<.0001	1.879	1.485–2.379
	80–84		<.0001	2.309	1.776–3.001
	85–90		< 0.001	2.257	1.545–3.298
	= > 90		<.0001	4.457	2.614–7.599
Gender					
	Male	Female	0.2969	1.124	0.903–1.399
Treatment type					
	CRT+/−surgery	RT +/− surgery	0.6839	1.053	0.821–1.350
	Surgery+/−RT/CRT		0.7389	1.034	0.851–1.256
Elixhauser Comorbidity Index Score					
	1	0	0.1105	1.194	0.960–1.484
	2		0.0618	1.298	0.987–1.708
	3+		<.0001	1.902	1.524–2.375
Primary site					
	Hypopharynx	Glottic larynx	<.0001	2.933	2.336–3.683
	Nasopharynx		0.0101	1.679	1.131–2.492
	Supraglottic larynx		<.0001	2.296	1.867–2.825
Metformin use					
	Control (no metformin exposure)	Case (metformin use at time of diagnosis)	0.4314	1.104	0.863–1.413

*CI* confidence interval, *RT* radiation therapy, *CRT* concurrent chemoradiation therapy

Results from multivariate analysis of covariates for DSS are summarized in Table 3. Metformin use was not associated with a significant improvement in DSS (*p* = 0.9980, HR 1.000, 95% CI [0.695–1.440]).

**Table 3:** Multivariate regression analysis for disease specific survival (DSS) in patients taking metformin at time of diagnosis

Covariate	Category	Comparator	*P*-value	Hazard Ratio	95% CI
Age					
	70–74	65–69	0.8138	0.958	0.673–1.364
	75–79		0.0035	1.636	1.175–2.277
	80–84		0.0067	1.726	1.163–2.561
	85–90		0.0450	1.793	1.013–3.175
	= > 90		0.0275	2.832	1.122–7.148
Gender					
	Male	Female	0.1428	1.283	0.919–1.791
Treatment type					
	CRT+/−surgery	RT +/− surgery	0.2510	1.218	0.870–1.704
	Surgery+/−RT/CRT		0.1351	1.247	0.933–1.667
Elixhauser Comorbidity Index Score					
	1	0	0.8172	1.040	0.743–1.457
	2		0.3511	1.218	0.805–1.842
	3+		0.0078	1.582	1.128–2.218
Primary site					
	Hypopharynx	Glottic larynx	<.0001	4.488	3.269–6.162
	Nasopharynx		< 0.001	2.957	1.757–4.977
	Supraglottic larynx		<.0001	2.993	2.189–4.093
Metformin use					
	Control (no metformin exposure)	Case (metformin use at time of diagnosis)	0.9980	1.000	0.695–1.440

*CI* confidence interval, *RT* radiation therapy, *CRT* concurrent chemoradiation therapy

Secondary analyses were performed in order to determine whether duration of metformin use impacted results. These were performed with adjusted case definitions of: (a) metformin use for a minimum of 1 year prior to diagnosis and a minimum of 4 months after diagnosis, and, (b) metformin use for a minimum of 1 month prior to diagnosis and a minimum of 4 months after diagnosis. These analyses were treated as secondary analyses as there is an inherent survival bias for those patients remaining on metformin after treatment (only patients who remained alive long enough to receive metformin after treatment could be examined). For each of these, there was no significant effect of metformin use on OS or DSS when compared to control patients not taking metformin for a minimum of 1 year before and 1 year after the time of diagnosis. (Additional file 1: Table S1, Additional file 2: Table S2, Additional file 3: Table S3 and Additional file 4: Table S4). In the first secondary analysis in which cases were defined as metformin use for a minimum of 1 year prior to and 4 months after diagnosis, after taking into account the aforementioned exclusions, 95 cases and 1011 metformin non-users were identified. There was no significant difference in OS (*p* = 3610, HR 1.168, 95% CI [0.837–1.630]) or DSS (*p* = 0.6822, HR 0.902, 95% CI [0.549–1.480]) between cases and metformin non-users. In the second scenario, in which cases were defined as metformin use for a minimum of 1 month prior to and 4 months after diagnosis, after taking into account the aforementioned exclusions, 118 cases and 1018 non-users were identified. There was again no significant difference in OS (*p* = 3639, HR 1.150, 95% CI [0.850 = 1.557]) or DSS (*p* = 0.9709, HR 0.991, 95% CI [0.623–1.578]).

## Discussion

Metformin has demonstrated anti-tumorigenic effects both in vitro and in vivo, and has been shown to improve treatment response in breast and colorectal cancers [26–32]. Metformin’s mechanism of action, biologically plausible mechanism of tumor suppression, and minimal side-effect profile make it an appealing anti-cancer therapy. While preliminary data exists suggesting that metformin may improve survival in patients with HNSCC of the larynx [35], our large-scale analysis has shown that no survival benefit exists from the use of metformin in squamous cell cancer of the larynx, nasopharynx, or hypopharynx. The data used in our multivariate analysis was compiled from all patients in Ontario diagnosed with these cancers over a 5-year period.

There have been several animal studies and one retrospective study in humans suggesting that metformin may improve survival outcomes for HNSCC patients [44]. Sandulache et al. conducted a large-scale retrospective cohort study to examine the association between metformin use and improved survival in cancers of the larynx [35]. In multivariate analysis, they demonstrated improved OS (*p* = 0.04) when comparing diabetics using metformin to diabetics not on metformin. However, they did not demonstrate an improvement in either OS or DFS when they compared non-diabetics to diabetics using metformin. It is difficult to determine if the improved survival in diabetics on metformin compared to diabetics not on metformin (which could include diabetics with complications such as nephropathy precluding metformin use) is actually representative of less severe diabetes in the metformin group rather than an improvement in survival as a result of metformin use. It stands to reason that those with more severe diabetes would have predictably worse OS compared with those whose sugars are controlled with the use of metformin. Our results are similar to Sandulache et al. in that when they compared non-diabetic metformin users to diabetics on metformin, they did not demonstrate an improvement in OS or DFS.

Limitations of our study include its retrospective design; a prospective study would be required in order to more rigorously assess for a survival advantage. The timeline of our study is such that survival data was available for all patients for a minimum of 3 full years following cancer diagnosis. We were therefore able to assess for changes in survival within this time-frame, but not long-term (five-year) survival for all patients in our cohort. We were unable to capture patients’ tumor/ node/ metastases (TNM) cancer staging at time of diagnosis. For cancers of the larynx and hypopharynx, we were able to infer staging based on treatment received – the distribution of which was equal between case and control groups – since treatment is largely stage-specific and is uniform across centres. Though stage-specific treatment protocols for nasopharygeal cancer may vary more across centres, it can also be argued that staging variability should not have been significantly different between case and control groups, since the decision to be on metformin at the time of HNSCC diagnosis is unrelated to cancer stage. We captured data relating to whether patients were taking metformin at the time of their cancer diagnosis, but could not ensure that they continued to use metformin throughout the study period; however, secondary analyses examining metformin use over alternative time periods similarly did not show significant results. We were also unable to capture data regarding smoking and alcohol history, as these are not available in our data sources. We also did not have data related to patient baseline performance status. As well, our study specifically assessed for a survival advantage of metformin use in patients with HPV-negative head and neck cancers. Since HPV status is not conventionally reported in most databases, including the data sources available to us, and due to its confounding effects on head and neck cancer survival, we focused our analysis on patients with cancers of the larynx, hypopharynx, and nasopharynx, which are typically HPV-negative cancers. We drew patient causes of death from the Office of the Registrar General Database (ORGD), where this information is extracted from death certificates. As death certificates are manually completed by physicians at the time of patient death, when there are often multiple disease processes at play, documented causes of death are not always reliable or replicable. This has the potential to impact our calculation of disease-specific survival. However, this would likely affect our calculation of DSS for both metformin users and non-users in a similar manner, since baseline demographic variables for both these groups were similar. Another inherent limitation in a study like ours is that it is impossible to exactly replicate the underlying disease states in the study and metformin non-use groups. The main indication for metformin treatment is diabetes and as such, comparing those on metformin to those not on metformin has the inherent difference that those patients on metformin will have underlying diabetes, contributing to a distinctly different disease state from those without.

## Conclusion

Our large scale retrospective cohort study demonstrated that patients taking metformin at the time of diagnosis of squamous cell carcinoma of the larynx, hypopharynx, and nasopharynx do not have improved survival compared with those not on metformin. This negative finding, which is in contrast to prior studies, highlights the need for further analysis in the form of a large-scale prospective study in order to reach a definitive conclusion. We would encourage such a study, as metformin’s biologically plausible mechanism of action and paucity of side effects make it an appealing and potentially efficacious novel therapeutic agent.

## Additional files

Additional file 1: **Table S1.** Multivariate regression analysis for overall survival (OS) in patients taking metformin for at least 1 year before diagnosis and 4 months after diagnosis. (DOCX 18 kb)

Additional file 2: **Table S2.** Multivariate regression analysis for disease specific survival (DSS) in patients taking metformin for at least 1 year before diagnosis and 4 months after diagnosis. (DOCX 18 kb)

Additional file 3: **Table S3.** Multivariate regression analysis for overall survival (OS) in patients taking metformin for at least 1 month before diagnosis and 4 months after diagnosis. (DOCX 19 kb)

Additional file 4: **Table S4.** Multivariate regression analysis for disease specific survival (DSS) in patients taking metformin for at least 1 month before diagnosis and 4 months after diagnosis. (DOCX 18 kb)

## Abbreviations

ALR: Activity Level Reporting
AMPK: AMP-activated protein kinase
CI: Confidence interval
CIHI: Canadian Institute for Health Information
CRT: Chemoradiotherapy
DSS: Disease-specific survival
HNSCC: Head and neck squamous cell carcinoma
HPV: Human Papillomavirus
HR: Hazards ratio
ODB: Ontario Drug Benefit Database
OHIP: Ontario Health Insurance Plan
ORGD: Office of the Registrar General Database
OS: Overall survival
RT: Radiation therapy
TNM: Tumor/node/metastases
