# Supplementary material for: Does metformin usage improve survival in head and neck squamous cell carcinoma? A population-based study
Source: J Otolaryngol Head Neck Surg. 2018 Dec 4;47:74. doi: 10.1186/s40463-018-0322-7 (PMC6278022; doi:10.1186/s40463-018-0322-7)
Supplement: Supplementary file 1 — Table S1. Multivariate regression analysis for overall survival (OS) in patients taking metformin for at least 1 year before diagnosis and 4 months after diagnosis. (DOCX 18 kb) [file 40463_2018_322_MOESM1_ESM.docx]

**Additional file 1: Table S1**. *Multivariate regression analysis for overall survival (OS) in patients taking metformin* *for at least 1 year before diagnosis and 4 months after diagnosis*

| *Covariate* | *Category* | *Comparator* | *P-value* | *Hazard Ratio* | *95% CI* |
| --- | --- | --- | --- | --- | --- |
| Age |  |  |  |  |  |
|  | 70-74 | 65-69 | 0.1404 | 1.220 | 0.937 – 1.590 |
|  | 75-79 |  | <.0001 | 1.954 | 1.511 – 2.528 |
|  | 80-84 |  | <.0001 | 2.302 | 1.723 – 3.076 |
|  | 85-90 |  | <0.001 | 2.415 | 1.624 – 3.593 |
|  | =>90 |  | <.0001 | 3.806 | 2.017 – 7.182 |
| Gender |  |  |  |  |  |
|  | Male | Female | 0.3601 | 1.118 | 0.881 – 1.418 |
| Treatment type | |  |  |  |  |
|  | CRT+/-surgery | RT +/- surgery | 0.4473 | 1.108 | 0.850 – 1.444 |
|  | Surgery+/-RT/CRT |  | 0.3997 | 0.912 | 0.736 – 1.130 |
| Elixhauser Comorbidity Index Score |  |  |  |  |  |
|  | 1 | 0 | 0.1104 | 1.209 | 0.958 – 1.528 |
|  | 2 |  | 0.0267 | 1.392 | 1.039 – 1.865 |
|  | 3+ |  | <.0001 | 1.910 | 1.492 – 2.446 |
| Primary site |  |  |  |  |  |
|  | Hypopharynx | Glottic larynx | <.0001 | 3.192 | 2.501 – 4.075 |
|  | Nasopharynx |  | 0.0245 | 1.641 | 1.066 – 2.525 |
|  | Supraglottic larynx |  | <.0001 | 2.266 | 1.810 – 2.837 |
| Metformin use |  |  |  |  |  |
|  | Control (no metformin exposure) | Case (metformin use 1 year before and 4 mo after diagnosis) | 0.3610 | 1.168 | 0.837 – 1.630 |

CI = confidence interval, RT = radiation therapy, CRT = concurrent chemoradiation therapy
