# Supplementary material for: Does metformin usage improve survival in head and neck squamous cell carcinoma? A population-based study
Source: J Otolaryngol Head Neck Surg. 2018 Dec 4;47:74. doi: 10.1186/s40463-018-0322-7 (PMC6278022; doi:10.1186/s40463-018-0322-7)
Supplement: Supplementary file 2 — Table S2. Multivariate regression analysis for disease specific survival (DSS) in patients taking metformin for at least 1 year before diagnosis and 4 months after diagnosis. (DOCX 18 kb) [file 40463_2018_322_MOESM2_ESM.docx]

**Additional file 2: Table S2**. *Multivariate regression analysis for disease specific survival (DSS) in patients taking metformin for at least 1 year before diagnosis and 4 months after diagnosis*

| *Covariate* | *Category* | *Comparison category* | *P-value* | *Hazard Ratio* | *95% CI* |
| --- | --- | --- | --- | --- | --- |
| Age |  |  |  |  |  |
|  | 70-74 | 65-69 | 0.9651 | 0.992 | 0.677 – 1.452 |
|  | 75-79 |  | 0.0078 | 1.630 | 1.137 – 2.338 |
|  | 80-84 |  | 0.1981 | 1.357 | 0.852 – 2.160 |
|  | 85-90 |  | 0.0727 | 1.758 | 0.949 – 3.255 |
|  | =>90 |  | 0.2089 | 2.127 | 0.656 – 6.901 |
| Gender |  |  |  |  |  |
|  | Male | Female | 0.0592 | 1.455 | 0.986 – 2.147 |
| Treatment type | |  |  |  |  |
|  | CRT+/-surgery | RT +/- surgery | 0.1350 | 1.318 | 0.918 – 1.893 |
|  | Surgery+/-RT/CRT |  | 0.5035 | 1.118 | 0.806 – 1.551 |
| Elixhauser Comorbidity Index Score |  |  |  |  |  |
|  | 1 | 0 | 0.9554 | 0.989 | 0.681 – 1.438 |
|  | 2 |  | 0.2722 | 1.288 | 0.820 – 2.024 |
|  | 3+ |  | 0.0572 | 1.469 | 0.988 – 2.182 |
| Primary site |  |  |  |  |  |
|  | Hypopharynx | Glottic larynx | <.0001 | 5.106 | 3.624 – 7.193 |
|  | Nasopharynx |  | 0.0013 | 2.664 | 1.464 – 4.848 |
|  | Supraglottic larynx |  | <.0001 | 2.777 | 1.951 – 3.953 |
| Metformin use |  |  |  |  |  |
|  | Control (no metformin exposure) | Case (metformin use 1 year before and 4 mo after diagnosis) | 0.6822 | 0.902 | 0.549 – 1.480 |

CI = confidence interval, RT = radiation therapy, CRT = concurrent chemoradiation therapy
