# Supplementary material for: Does metformin usage improve survival in head and neck squamous cell carcinoma? A population-based study
Source: J Otolaryngol Head Neck Surg. 2018 Dec 4;47:74. doi: 10.1186/s40463-018-0322-7 (PMC6278022; doi:10.1186/s40463-018-0322-7)
Supplement: Supplementary file 3 — Table S3. Multivariate regression analysis for overall survival (OS) in patients taking metformin for at least 1 month before diagnosis and 4 months after diagnosis. (DOCX 19 kb) [file 40463_2018_322_MOESM3_ESM.docx]

**Additional file 3: Table S3**. *Multivariate regression analysis for overall survival (OS) in patients taking metformin* *for at least 1 month before diagnosis and 4 months after diagnosis*

| *Covariate* | *Category* | *Comparator* | *P-value* | *Hazard Ratio* | *95% CI* |
| --- | --- | --- | --- | --- | --- |
| Age |  |  |  |  |  |
|  | 70-74 | 65-69 | 0.1182 | 1.230 | 0.949 – 1.595 |
|  | 75-79 |  | <.0001 | 1.951 | 1.518 – 2.509 |
|  | 80-84 |  | <.0001 | 2.291 | 1.723 – 3.046 |
|  | 85-90 |  | <0.001 | 2.431 | 1.639 – 3.607 |
|  | =>90 |  | <.0001 | 3.823 | 2.031 – 7.198 |
| Gender |  |  |  |  |  |
|  | Male | Female | 0.3464 | 1.120 | 0.884 – 1.419 |
| Treatment type | |  |  |  |  |
|  | CRT+/-surgery | RT +/- surgery | 0.7150 | 1.050 | 0.807 – 1.367 |
|  | Surgery+/-RT/CRT |  | 0.4461 | 0.921 | 0.745 – 1.138 |
| Elixhauser Comorbidity Index Score |  |  |  |  |  |
|  | 1 | 0 | 0.1521 | 1.184 | 0.940 – 1.492 |
|  | 2 |  | 0.0334 | 1.365 | 1.025 – 1.818 |
|  | 3+ |  | <.0001 | 1.819 | 1.424 – 2.323 |
| Primary site |  |  |  |  |  |
|  | Hypopharynx | Glottic larynx | <.0001 | 3.204 | 2.516 – 4.081 |
|  | Nasopharynx |  | 0.0288 | 1.615 | 1.051 – 2.482 |
|  | Supraglottic larynx |  | <.0001 | 2.266 | 1.812 – 2.834 |
| Metformin use |  |  |  |  |  |
|  | Control (no metformin exposure) | Case (metformin use 1 mo before and 4 mo after diagnosis) | 0.3639 | 1.150 | 0.850 – 1.557 |

CI = confidence interval, RT = radiation therapy, CRT = concurrent chemoradiation therapy
