# Supplementary material for: Does metformin usage improve survival in head and neck squamous cell carcinoma? A population-based study
Source: J Otolaryngol Head Neck Surg. 2018 Dec 4;47:74. doi: 10.1186/s40463-018-0322-7 (PMC6278022; doi:10.1186/s40463-018-0322-7)
Supplement: Supplementary file 4 — Table S4. Multivariate regression analysis for disease specific survival (DSS) in patients taking metformin for at least 1 month before diagnosis and 4 months after diagnosis. (DOCX 18 kb) [file 40463_2018_322_MOESM4_ESM.docx]

**Additional file 4: Table S4**. *Multivariate regression analysis for disease specific survival (DSS) in patients taking metformin for at least 1 month before diagnosis and 4 months after diagnosis*

| *Covariate* | *Category* | *Comparison category* | *P-value* | *Hazard Ratio* | *95% CI* |
| --- | --- | --- | --- | --- | --- |
| Age |  |  |  |  |  |
|  | 70-74 | 65-69 | 0.7434 | 1.065 | 0.730 – 1.553 |
|  | 75-79 |  | 0.0028 | 1.722 | 1.206 – 2.458 |
|  | 80-84 |  | 0.1536 | 1.402 | 0.881 – 2.230 |
|  | 85-90 |  | 0.0511 | 1.845 | 0.997 – 3.414 |
|  | =>90 |  | 0.1759 | 2.253 | 0.695 – 7.305 |
| Gender |  |  |  |  |  |
|  | Male | Female | 0.0838 | 1.403 | 0.956 – 2.060 |
| Treatment type | |  |  |  |  |
|  | CRT+/-surgery | RT +/- surgery | 0.2142 | 1.256 | 0.877 – 1.800 |
|  | Surgery+/-RT/CRT |  | 0.5295 | 1.110 | 0.802 – 1.537 |
| Elixhauser Comorbidity Index Score |  |  |  |  |  |
|  | 1 | 0 | 0.0.8221 | 0.958 | 0.660 – 1.392 |
|  | 2 |  | 0.2658 | 1.282 | 0.828 – 1.986 |
|  | 3+ |  | 0.1157 | 1.374 | 0.925 – 2.042 |
| Primary site |  |  |  |  |  |
|  | Hypopharynx | Glottic larynx | <.0001 | 5.103 | 3.632- 7.171 |
|  | Nasopharynx |  | 0.0016 | 2.606 | 1.436 – 4.732 |
|  | Supraglottic larynx |  | <.0001 | 2.794 | 1.967 – 3.969 |
| Metformin use |  |  |  |  |  |
|  | Control (no metformin exposure) | Case (metformin use 1 mo before and 4 mo after diagnosis) | 0.9709 | 0.991 | 0.623 – 1.578 |

CI = confidence interval, RT = radiation therapy, CRT = concurrent chemoradiation therapy
